# Spatiotemporal Analysis of BTEX and PM Using Me-DOAS and GIS in Busan’s Industrial Complexes

**DOI:** 10.3390/toxics13080638

**Published:** 2025-07-29

**Authors:** Min-Kyeong Kim, Jaeseok Heo, Joonsig Jung, Dong Keun Lee, Jonghee Jang, Duckshin Park

**Affiliations:** 1 R&D Strategy Department, Korea Railroad Research Institute (KRRI), Cheoldo Bangmulgwanro, Uiwang-si 16105, Republic of Korea; mkkim15@krri.re.kr; 2 Transportation Environmental Research Department, Korea Railroad Research Institute (KRRI), Cheoldo Bangmulgwanro, Uiwang-si 16105, Republic of Korea; jsheo1005@krri.re.kr; 3 Nakdong River Basin Environment Office, Changwon-si 51439, Republic of Korea; jsjung080@korea.kr (J.J.); ldk0225@korea.kr (D.K.L.); jhjang1013@korea.kr (J.J.)

**Keywords:** 3D geographic information system (GIS), hotspot analysis, solar occultation flux (SOF), mobile extractive differential optical absorption spectroscopy (Me-DOAS), Busan industrial complex, BTEX, volatile organic compounds (VOCs)

## Abstract

Rapid industrialization and urbanization have progressed in Korea, yet public attention to hazardous pollutants emitted from industrial complexes remains limited. With the increasing coexistence of industrial and residential areas, there is a growing need for real-time monitoring and management plans that account for the rapid dispersion of hazardous air pollutants (HAPs). In this study, we conducted spatiotemporal data collection and analysis for the first time in Korea using real-time measurements obtained through mobile extractive differential optical absorption spectroscopy (Me-DOAS) mounted on a solar occultation flux (SOF) vehicle. The measurements were conducted in the Saha Sinpyeong–Janglim Industrial Complex in Busan, which comprises the Sasang Industrial Complex and the Sinpyeong–Janglim Industrial Complex. BTEX compounds were selected as target volatile organic compounds (VOCs), and real-time measurements of both BTEX and fine particulate matter (PM) were conducted simultaneously. Correlation analysis revealed a strong relationship between PM_10_ and PM_2.5_ (r = 0.848–0.894), indicating shared sources. In Sasang, BTEX levels were associated with traffic and localized facilities, while in Saha Sinpyeong–Janglim, the concentrations were more influenced by industrial zoning and wind patterns. Notably, inter-compound correlations such as benzene–m-xylene and p-xylene–toluene suggested possible co-emission sources. This study proposes a GIS-based, three-dimensional air quality management approach that integrates variables such as traffic volume, wind direction, and speed through real-time measurements. The findings are expected to inform effective pollution control strategies and future environmental management plans for industrial complexes.

## 1. Introduction

South Korea has rapidly industrialized over the past few decades, resulting in the development of 1330 industrial complexes as of 2024, including 53 national complexes and numerous general, urban high-tech, and agro-industrial zones, which collectively employ about 2.37 million workers [1]. Due to the country’s limited land availability, residential developments have increasingly been situated adjacent to industrial zones, intensifying concerns over environmental pollution and health risks [2,3]. Although public awareness of particulate matter (PM) and offensive odors has increased, hazardous air pollutants (HAPs)—particularly volatile organic compounds (VOCs)—remain relatively under-recognized, despite their significant health impacts. Exposure to VOCs has been associated with respiratory diseases, neurological damage, and reproductive disorders, and VOCs also contribute to the formation of secondary pollutants such as ozone and PM through atmospheric chemical reactions [3,4,5]. The Korean Clean Air Conservation Act currently designates 35 HAPs, including benzene and ethylbenzene, as controlled substances; however, air quality standards for many other compounds have not yet been established [6,7].

In 2022, Korea’s total emissions included 59,459 tons of PM_2.5_ and 938,341 tons of VOCs, with the latter primarily originating from solvent use (54.9%) and industrial processes (15.5%) [8]. While emissions have generally decreased since 2016 due to regulatory improvements and cleaner technologies, the detailed real-time monitoring of VOCs remains limited, especially at the facility level [9]. Most existing studies rely on fixed monitoring stations, which fail to adequately capture the dynamic and spatial variability of airborne pollutants influenced by traffic patterns, meteorology, and land use. Previous studies in Busan have examined pollutant levels in industrial areas [10,11,12,13,14,15,16,17,18], but most relied on fixed monitoring stations or data from local authorities. These stationary methods are limited in capturing the spatiotemporal variability of pollutants influenced by factors such as wind and traffic. Although mobile measurements help to address these limitations, they also face challenges such as short campaign durations, potential measurement bias due to vehicle motion, and difficulty in correcting background concentrations or identifying specific sources. Nonetheless, GIS-based visualization techniques can mitigate some of these issues through the integration of environmental and anthropogenic variables to map pollutant influence spatially. Recent research has increasingly adopted mobile monitoring to study VOC distributions over time and space [19,20,21,22,23,24,25,26]. This study differs in that it applies mobile measurements and GIS-based 3D spatial analysis, incorporating not only VOC concentrations but also contextual variables such as traffic volume. Traditional VOC analysis methods—such as adsorbent tubes, Tedlar bags, and GC–MS or HPLC—suffer from time delays and limited temporal resolution. In contrast, real-time techniques such as proton transfer reaction mass spectrometry (PTR-MS) and selective ion flow tube mass spectrometry (SIFT-MS) offer high temporal resolution and the ability to detect VOCs at ppb levels [7,27,28]. Among these, SIFT-MS enables direct, real-time quantification of trace gases without the need for pre-concentration. However, SIFT-MS still faces limitations, such as high background signal, sensitivity to water vapor, and challenges in distinguishing isomeric compounds, which necessitate careful calibration and strict quality control procedures [29]. Compared to these techniques, Me-DOAS offers a different approach based on optical remote sensing, providing integrated path-averaged concentrations over large distances without direct sampling. While its sensitivity may be lower than SIFT-MS in detecting specific low-concentration compounds, Me-DOAS has clear advantages in spatial coverage, robustness for mobile deployment, and suitability for atmospheric plume characterization.

To address these challenges, this study used mobile extractive differential optical absorption spectroscopy (Me-DOAS) mounted on a solar occultation flux (SOF) vehicle [30]. Me-DOAS enables real-time VOC detection using ultraviolet light without pretreatment and, when combined with meteorological sensors, allows for comprehensive spatiotemporal analyses. Though Me-DOAS is sensitive to low absorbance and background variation, these are managed through stabilization procedures, reference point selection, and signal-to-noise evaluation to ensure data reliability.

Accordingly, this study aims to quantify the spatiotemporal distribution patterns of VOC emissions in industrial complexes by integrating real-time mobile monitoring data with three-dimensional GIS-based spatial analysis. Specifically, the study seeks to identify high-concentration zones, evaluate correlations with traffic and land-use factors, and delineate emission patterns over time and space. Focusing on two representative industrial areas in Busan—Sasang, with high traffic congestion, and Sinpyeong–Janglim, with comparatively lower traffic intensity—this study investigates how variations in traffic volume influence ambient VOC concentrations under similar regional and environmental conditions. Particular attention is given to the spatial and temporal distribution of emissions, the role of traffic-related activity, and the interaction with meteorological and geographic variables, such as wind direction and site layout. This study hypothesizes that traffic density serves as a significant modulating factor of ambient VOC concentrations, even within industrial zones exhibiting comparable emission profiles. It further posits that the observed spatiotemporal variability in VOC levels arises from the interplay among traffic-related emissions, localized industrial discharge characteristics, atmospheric dispersion dynamics, and temporal fluctuations in emission activity. By employing mobile extractive differential optical absorption spectroscopy (Me-DOAS) mounted on a solar occultation flux (SOF) vehicle and combining the resulting data with contextual environmental parameters, this study seeks to establish a robust, data-driven framework for air quality management in complex industrial environments.

## 2. Materials and Methods

### 2.1. Research Site and Measurement Procedure

In this study, the Sasang Industrial Complex and the Saha Sinpyeong–Janglim Industrial Complex in Busan were selected as target sites for air quality monitoring. These sites were chosen due to their need for air pollution management and the availability of relevant prior studies, rendering them suitable for comparative analysis.

**Figure 1:**
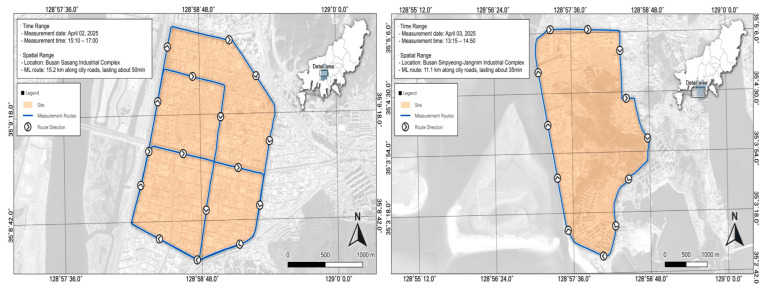
Study site locations and SOF (solar occultation flux) vehicle measurement routes.

Mobile measurements were conducted within both industrial complexes (Figure 1). The survey followed predefined routes, and all equipment mounted on the solar occultation flux (SOF) vehicle was stabilized prior to operation. The measurement route in the Sasang Industrial Complex was approximately 12.1 km in length and took about 50 min per run due to frequent traffic congestion. In contrast, the route in the Saha Sinpyeong–Janglim Industrial Complex was about 11.8 km and required approximately 35 min to complete due to lighter traffic conditions. To establish baseline comparisons, Macdo Park near the Sasang complex and Dadae Riverside Park near the Saha complex were selected as reference areas.

The field campaign was conducted over two weekdays—2 and 3 April—during the spring season. In total, ten measurement runs were completed: four on 2 April (9:00–11:00 a.m. and 2:00–4:00 p.m.) and six on 3 April (9:00–11:00 a.m. and 1:00–3:00 p.m.). Each industrial complex—Sasang and Saha Sinpyeong–Janglim—was surveyed for approximately 4 to 5 h in total, with mobile transects conducted during both morning and afternoon sessions to capture diurnal variations in traffic and meteorological conditions. It should be noted that night-time boundary layer dynamics and weekend industrial shift operations were not included in the survey scope.

The vehicle maintained a constant speed of approximately 20 km/h throughout data collection. During the measurements, the wind speed was recorded at 1.9 m/s at the Sasang site and 5.2 m/s at the Saha Sinpyeong–Janglim site. In this study, low-speed driving at approximately 20 km/h was maintained in major sections to minimize fluctuations in vehicle speed. Measurements were conducted during periods of relatively stable traffic and weather conditions, and potential bias was reduced through repeated measurements. Furthermore, temporary disturbances were addressed by applying outlier removal techniques and correcting for background concentrations.

Although mobile measurements are based on short-term campaigns and may have limitations in representing long-term air quality conditions, they offer valuable insight into the spatial distribution and relative trends in pollutant concentrations across different areas. In this study, measurements were conducted under consistent meteorological conditions and traffic patterns to minimize temporal variability. Furthermore, by comparing areas with similar regional characteristics, the analysis was able to identify spatial patterns and correlations with environmental variables, such as traffic volume. While long-term fixed monitoring is essential for trend analysis, short-term mobile measurements provide complementary data that can reveal localized pollution hotspots and guide future monitoring strategies.

### 2.2. Experimental Methods

#### 2.2.1. Measuring Vehicles and Equipment

**Figure 2:**
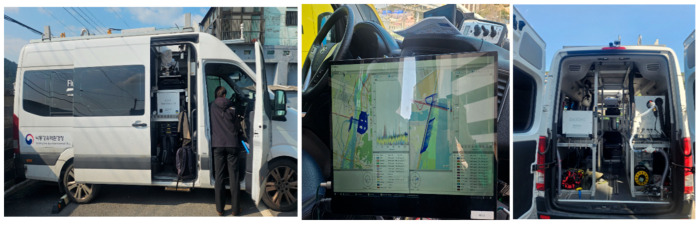
Me-DOAS measurement system mounted on the SOF (solar occultation flux) vehicle.

In this study, a mobile extractive differential optical absorption spectroscopy (Me-DOAS) system (FluxSense Co., Ltd., Göteborg, Sweden) was installed on a solar occultation flux (SOF) vehicle for real-time atmospheric monitoring (Figure 2). Me-DOAS is an optical device used to measure volatile organic compounds (VOCs), including BTEX (benzene, toluene, ethylbenzene, and xylenes), by utilizing ultraviolet (UV) absorption. The system employs a White cell mirror configuration with a total optical path length of 210 m, operating within a UV wavelength range of 255–285 nm. VOC analysis is performed using a DOAS spectrometer. The device has a detection limit of 0.2–3 ppb, with an accuracy of ±10% and a precision of ±5%. To ensure the accuracy of the measurements, the vehicle maintained a driving speed below 20 km/h. As monitoring was performed using a diesel-powered vehicle and the measurement inlet was installed on the rooftop, potential interference from the vehicle’s exhaust emissions was negligible. Real-time GPS data (including location and time) were continuously recorded and synchronized with mapping data provided by FluxSense.

At present, only two Me-DOAS units are in operation in Korea, and no official domestic inspection or calibration system exists. However, Me-DOAS offers the advantage of real-time source tracking by comparing the measurement spectrum with the background spectrum, a capability inherent to the ORS device. Moreover, to ensure data reliability, it regularly monitors for abnormalities in the spectrum, absorbance, background signals, light source stability, and laser path alignment, making it a highly dependable instrument. The Me-DOAS equipment undergoes regular QA/QC inspections conducted by FluxSense and domestic engineering partners (BK Instruments, Yorba Linda, CA, USA). Key parameters—such as the light source, spectral data, and signal-to-noise ratio (SNR)—are routinely evaluated. A higher SNR indicates greater measurement accuracy and reliability, whereas a lower SNR results in increased noise, making it difficult to distinguish true signals from background interference. As the internal light source operates with a fixed optical path, the light path may become misaligned during movement; therefore, periodic inspections are conducted to maintain measurement stability.

Additionally, previous analyses using conventional DOAS techniques and GC-FID canister sampling have shown consistent results, with less than 10% error in BTEX concentrations [30]. During the 2013 DISCOVER-AQ campaign in Houston, USA, Me-DOAS results were also found to be in good agreement with those obtained from proton transfer reaction mass spectrometry (PTR-MS) and Me-DOAS [31]. In accordance with the manufacturer’s recommended procedures, the system was stabilized for over 40 min on an upwind baseline before data collection to ensure the stability of the light source and eliminate background noise fluctuations. PM concentration data were collected using a GRIMM 11-D dust monitor, which operates at a standard flow rate of 1.2 L/min. The instrument was calibrated according to the manufacturer’s guidelines, and a gravimetric correction factor was applied based on reference filter measurements. To ensure data quality, measurements were filtered based on spectral residual thresholds provided by the instrument’s software, and outliers exceeding three standard deviations from the median were excluded. Additionally, data points with particle counts below the detection limit or with signal instability (based on real-time spectrum diagnostics) were removed to minimize noise and ensure accuracy.

Raw data collected from the mobile measurements were matched with real-time GPS mapping data. All equipment was time-synchronized, and the measured pollutant data were integrated with GPS data to enable spatial analysis. To eliminate the time lag between the GRIMM 11-D PM monitor and the Me-DOAS VOC analyzer, post-synchronization was performed based on time metadata, and any discrepancies exceeding ±1 s were corrected using linear interpolation. This ensured accurate temporal alignment of spatial and concentration data across all sensors. Secondary processed data were then derived by removing anomalies, including stop points and abnormal segments. VOC concentration values were extracted for each valid geographic location. To remove outliers from the raw data, the following steps were applied: First, when negative values were observed, they were interpreted as resulting from a decreased SNR in low-concentration areas and considered below the minimum detection limit; such values were replaced with zero. Next, if a concentration spike occurred in a specific section and the source of contamination could not be identified, the data were excluded. Sudden fluctuations in concentration without a clear cause—especially when showing irregular high and low values—were regarded as low-reliability data. In such cases, the equipment was inspected, and the preheating and cooling states were reviewed to identify potential malfunctions. However, if the elevated concentration values were continuous rather than instantaneous, they were retained and treated as valid data.

#### 2.2.2. Target Substances

In this study, data were collected on several volatile organic compounds (VOCs), including fine particulate matter (PM), benzene, toluene, ethylbenzene, and m,p-xylene. These compounds were measured using two instruments: GRIMM 11-4D (Grimm Aerosol Technik, Berlin, Germany) and the Me-DOAS system. Both instruments were installed on the SOF (solar occultation flux) vehicle. The GRIMM system recorded data at 6 s intervals, while the Me-DOAS system collected measurements every 2 s (Table 1 and Table 2). 

Me-DOAS is an internal light source ORS instrument, and when measuring BTEX compounds, spectral overlaps can pose a challenge. Overlaps may occur with NO_2_, HCHO, and O_3_, which exhibit strong absorption in the ultraviolet region. In particular, toluene and xylene have broad and gentle absorption peaks that overlap with NO_2_, rendering accurate quantification difficult. Benzene, by contrast, is the most suitable compound for measurement due to its relatively low spectral interference. To address these issues, a reference area with minimal pollution sources in the surrounding environment was selected to stabilize the instrument and improve measurement reliability.

### 2.3. GIS Analysis

To analyze the spatiotemporal distribution of air pollutants in the Sasang and Saha Sinpyeong–Janglim Industrial Complexes in Busan, we utilized the open-source software QGIS (version 3.32). The GIS analysis was conducted using GPS-based coordinates from the SOF vehicle, along with time-resolved pollutant concentration data. Most VOCs are emitted in gaseous form, and the use of organic solvents—particularly in painting processes—represents the largest share of VOC emissions. Another significant source is vehicle-related pollutants, which are released during driving. Given that automobiles are also a major contributor to fine dust, traffic volume data were collected and incorporated into the analysis as a key variable. Traffic data were sourced from View-T, a national transport database maintained by the Korea Transport Institute.

## 3. Results

### 3.1. Concentration Distribution of PM and VOCs

The measurements were conducted in spring. On 2 April, the average temperature was 10.6 °C, with a wind speed of 1.9 m/s. On 3 April, the temperature was 10.1 °C, and wind speeds exceeded 5 m/s. The SOF vehicle measurement routes in both the Sasang and Saha Industrial Complexes passed through mixed residential and industrial areas. Table 3 summarizes the average pollutant concentrations measured during the mobile runs, and Table 4 presents the time-specific average values. Measurements were conducted during both morning and afternoon sessions on 2 and 3 April. However, only the stabilized afternoon values were used for analysis and interpretation. The sample size (N) for PM_10_ and PM_2.5_ measurements was 2675 on 2 April and 1233 on 3 April. For BTEX compounds, a total of 2201 data points were collected on 2 April, and 3304 were collected on 3 April.

On 2 April, the daily average PM concentrations from the Hakjang-dong monitoring station (near Sasang) were 27 µg/m^3^ for PM_10_ and 14 µg/m^3^ for PM_2.5_. On 3 April, the Janglim-dong station (near Saha) recorded 22 µg/m^3^ for PM_10_ and 11 µg/m^3^ for PM_2.5_. The mobile measurements for Sasang were consistent with the stationary station data, whereas the measurements for Saha were slightly higher than the daily average. PM_10_ and PM_2.5_ concentrations were not particularly high at either site.

Among VOCs, BTEX compounds were dominant. In Sasang, the BTEX composition was as follows: m-xylene (45.9%), ethylbenzene (31.7%), and toluene (17.9%). In Saha, the distribution was as follows: m-xylene (40.4%), toluene (25.5%), and ethylbenzene (12.7%).

The afternoon concentrations reflect localized factors. In Sasang, heavy traffic congestion and ongoing underground construction contributed to consistent emissions throughout the day, and this was unrelated to commuting hours. In contrast, traffic congestion in the Saha complex had a minimal impact on BTEX concentrations. BTEX compound dominance exhibited the following pattern: Sasang: m-xylene > ethylbenzene > toluene; Saha: m-xylene > toluene > ethylbenzene.

Control comparisons were made using nearby reference areas: Macdo Park (Sasang) and Dadae Riverside Park (Saha). In Sasang, benzene was half the level found in the target area, whereas toluene was 1.8 times higher, likely due to the prevailing wind direction (e.g., benzene: 0.9 µg/m^3^; toluene: 22.9 µg/m^3^; p-xylene: 5.0 µg/m^3^; m-xylene: 0 µg/m^3^; ethylbenzene: 0 µg/m^3^). In Saha, the control area exhibited generally low concentrations (e.g., benzene: 0 µg/m^3^; toluene: 11.1 µg/m^3^; p-xylene: 8.7 µg/m^3^; m-xylene: 14.2 µg/m^3^; ethylbenzene: 0 µg/m^3^).

Despite seasonal differences and temporal gaps, xylene and toluene were consistently detected at high levels in Sasang, likely due to the ongoing operations of small-scale facilities. Likewise, in Saha, BTEX concentrations (benzene, ethylbenzene, toluene, and xylene) were in line with past studies. However, unlike this study, previous research did not incorporate mobile emission sources or spatial variables and therefore could not identify spatial hotspots or location-specific influences.

### 3.2. The Correlation Between Pollutants and Traffic Congestion

Prior to performing Pearson correlation analysis, the normality of each variable distribution was assessed using the Shapiro–Wilk test and visual inspection through Q-Q plots. Most variables exhibited near-normal distribution patterns, validating the use of parametric correlation analysis. For variables that deviated slightly from normality (*p* < 0.05), the robustness of Pearson’s method was considered acceptable due to the sufficiently large number of observations and the exploratory nature of the analysis. Correlation coefficients were interpreted based on conventional thresholds (weak: |r| < 0.3; moderate: 0.3 ≤ |r| < 0.7; strong: |r| ≥ 0.7), and their significance was evaluated at the 95% confidence level.

Based on the collected data, the correlation analysis of the Sasang Industrial Complex revealed a strong relationship between PM_10_ and PM_2.5_ (r = 0.848). Among the pollutants, toluene exhibited a positive correlation (r = 0.175) with the duration of traffic congestion, indicating its association with traffic-related emissions. In contrast, benzene, xylenes, and ethylbenzene showed weak or negative correlations with traffic congestion, implying the involvement of different emission sources. Notably, m-xylene and benzene demonstrated a high correlation (r = 0.650), suggesting a potential common origin. These findings indicate that VOCs in the Sasang Industrial Complex may originate from a variety of sources, including industrial activities beyond traffic emissions (Table 5).

The correlation analysis of the Saha Sinpyeong–Janglim Industrial Complex revealed a strong relationship between PM_10_ and PM_2.5_ (r = 0.894), which may reflect influences from common emission sources such as industrial combustion or vehicular activity. However, it is important to note that such correlations can also be affected by atmospheric processes, including differences in chemical reactivity, dilution rates, and secondary aerosol formation. Toluene showed a weak positive correlation with traffic congestion (r = 0.125), while other BTEX components generally exhibited weak negative correlations. Notably, p-xylene and toluene (r = 0.442), as well as benzene and m-xylene (r = 0.483), showed moderate to strong correlations, suggesting the possibility of common emission sources. Ethylbenzene exhibited negative correlations with most variables, indicating distinct emission characteristics. These results suggest that the BTEX concentrations in this area may be more influenced by industrial activities than by traffic-related emissions (Table 6).

### 3.3. Spatiotemporal Patterns and Pollution Hotspot Analysis of PM and VOC Concentrations in Sasang Industrial Complex

In this study, concentrations of PM and VOCs in the Sasang Industrial Complex were measured along the SOF vehicle’s route and visualized as a three-dimensional (3D) spatial map using GIS. The GRIMM equipment was mounted on the SOF vehicle, with the inlet positioned at the top to sample ambient air. GPS data were integrated with pollutant concentration data to produce spatial visualizations.

**Figure 3:**
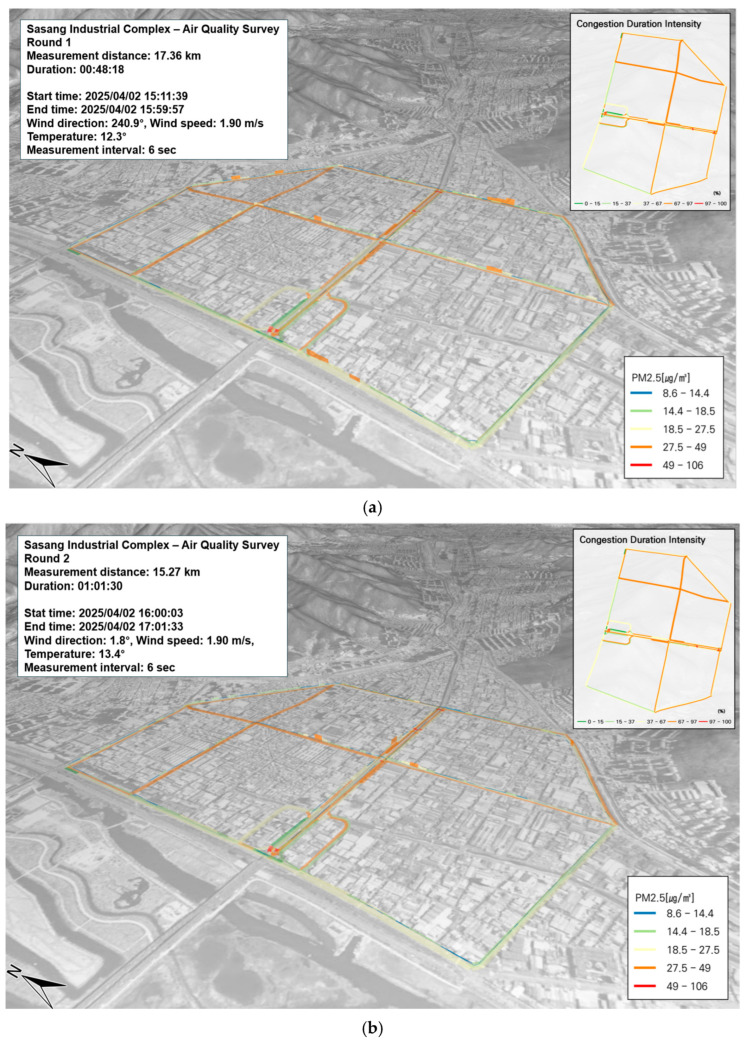

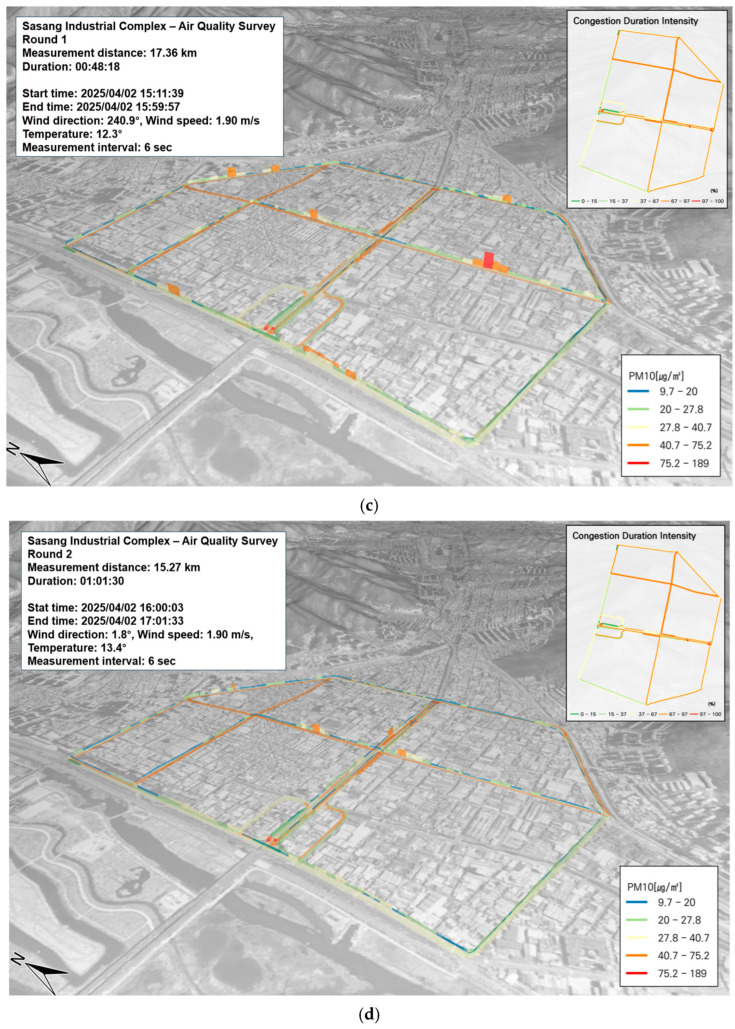
Spatial and temporal distribution of PM_2.5_ and PM_10_ concentrations on 2 April: (**a**) PM_2.5_, first run; (**b**) PM_2.5_, second run; (**c**) PM_10_, first run; (**d**) PM_10_, second run.

This study utilized GIS to derive results that reflect spatiotemporal characteristics according to the time of day. PM_10_ and PM_2.5_ were recorded at 6 s intervals. Given that fine particulate matter is strongly associated with road traffic, traffic congestion levels along the measurement route were compared with PM levels to identify pollution hotspots. No significant hotspots were observed for PM_2.5_. However, PM_10_ concentrations were notably higher in areas with severe traffic congestion, as illustrated in Figure 3c. This elevated PM_10_ level is believed to result from ongoing underground construction beneath the roadway. Ventilation openings installed at the center of the road likely allowed pollutants generated from both above-ground and underground activities to escape and contribute to the local PM_10_ concentration.

**Figure 4:**
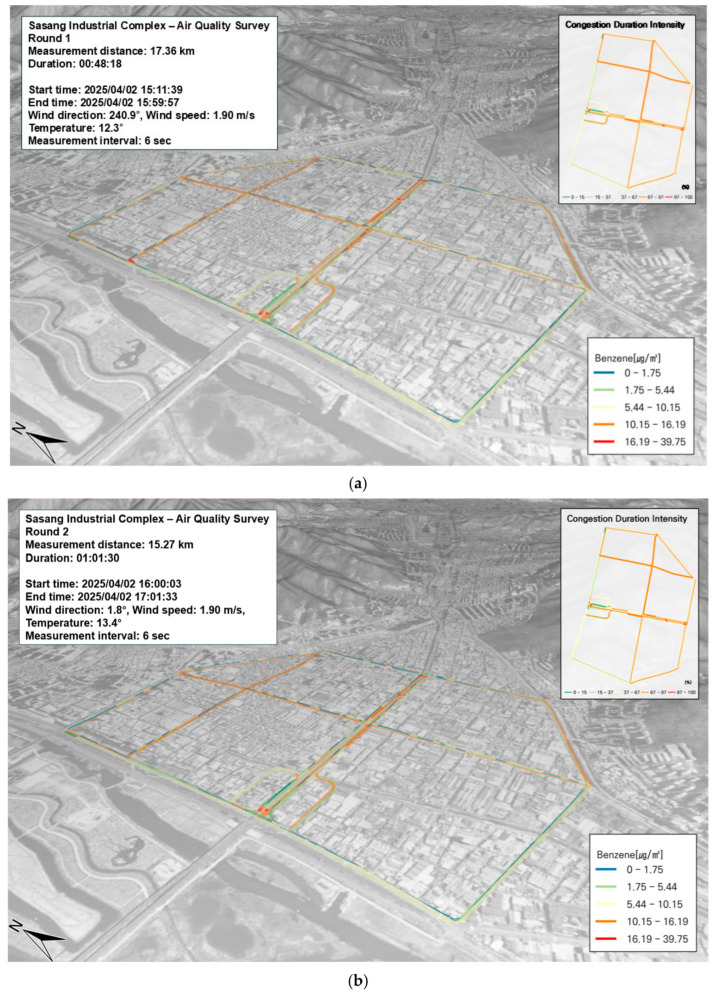

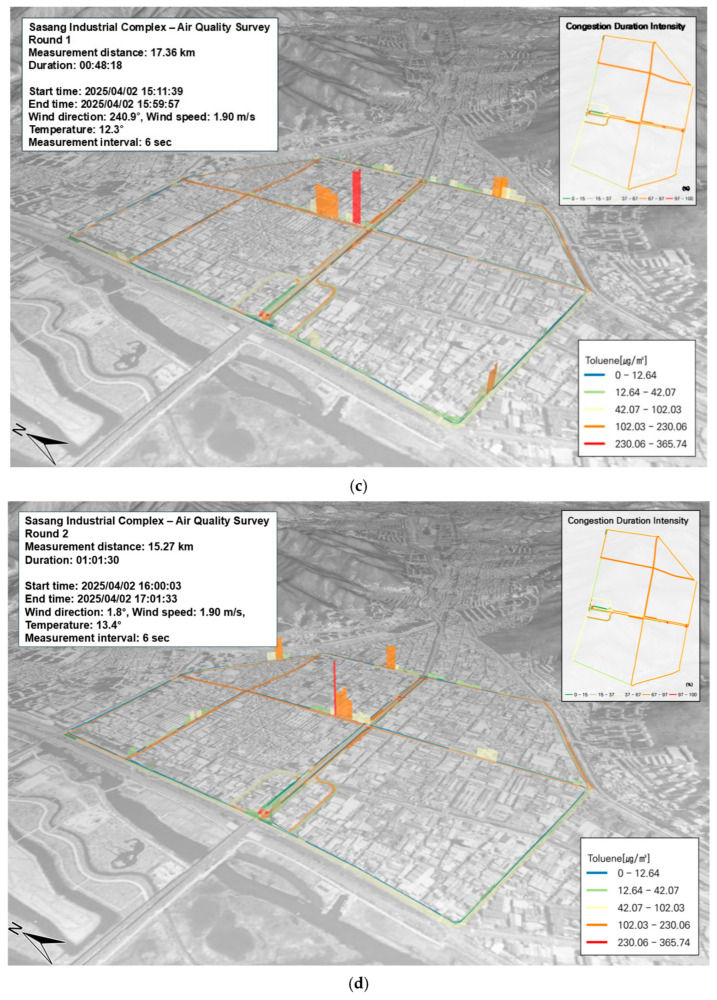

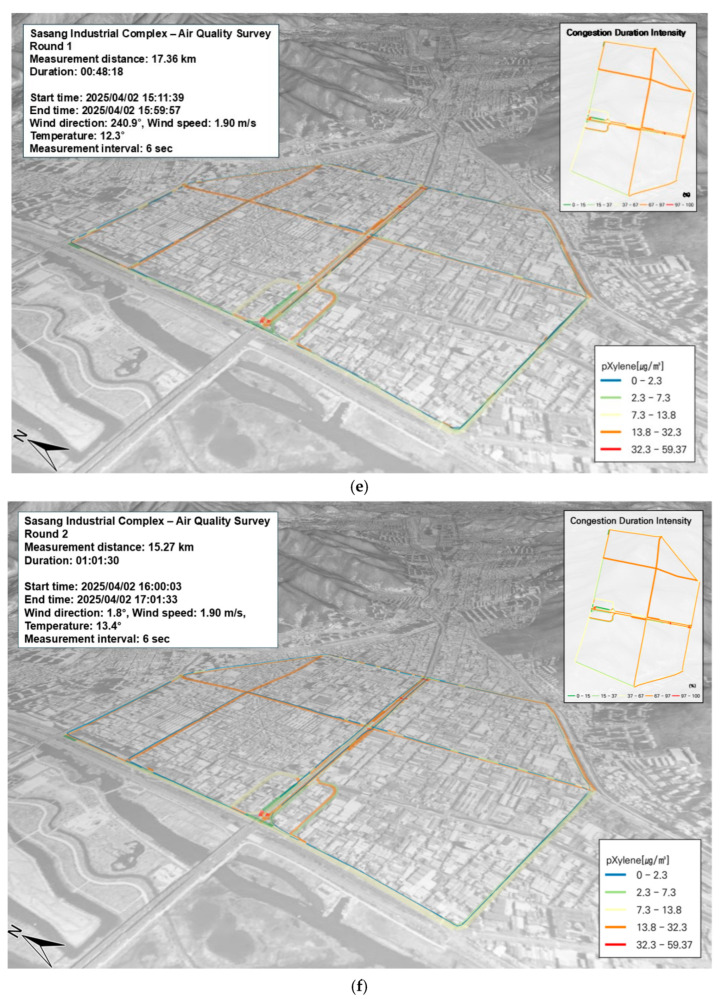

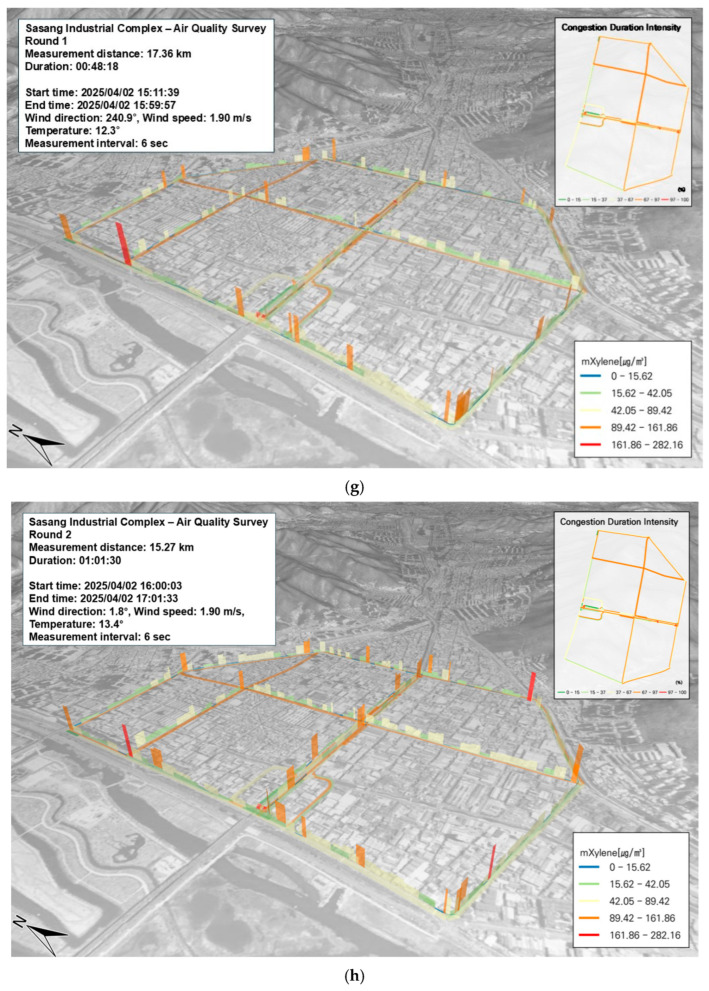

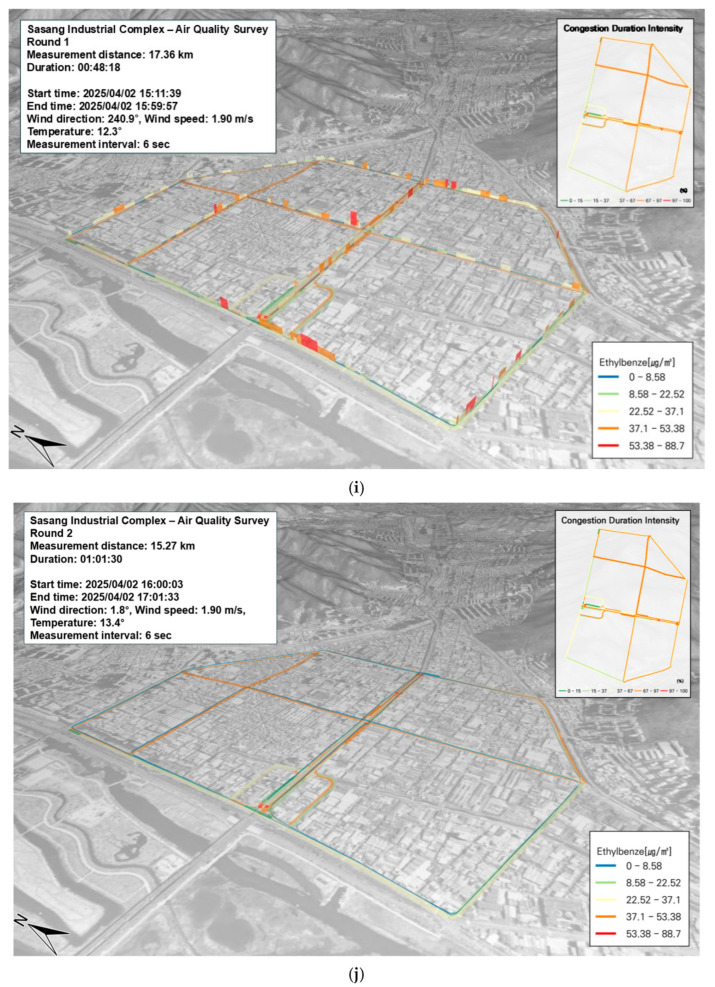
Temporal and spatial distribution of VOCs (BTEX) from mobile measurements conducted on 2 April: (**a**) benzene, first run; (**b**) benzene, second run; (**c**) toluene, first run; (**d**) toluene, second run; (**e**) p-Xylene, first run; (**f**) p-Xylene, second run; (**g**) m-Xylene, first run; (**h**) m-Xylene, second run; (**i**) ethylbenzene, first run; (**j**) ethylbenzene, second run.

In this study, benzene was selected as a representative compound among BTEX species for targeted analysis. According to the current air quality standard, the annual average concentration limit for benzene is 1.5 ppb. Benzene is a highly volatile compound that disperses rapidly in the atmosphere and is primarily absorbed through inhalation. As a known carcinogen, benzene exposure at high concentrations has been linked to hematologic malignancies, including leukemia. Short-term exposure can affect the central nervous system, causing symptoms such as dizziness, headache, confusion, loss of consciousness, and irritation of the eyes and skin. Long-term exposure may result in anemia by reducing both white and red blood cell counts and impairing immune function [19]. During the measurement period, the highest observed benzene concentration was 25.38 µg/m^3^, and this was recorded in the Sasang Industrial Complex. The average concentration across the site was 1.8 ± 2.7 µg/m^3^. No distinct benzene hotspots were detected along the entire route. When the benzene distribution was compared with traffic congestion data, no strong spatial correlation was observed. In fact, areas with high traffic volumes did not coincide with areas of elevated benzene concentrations (Figure 4a,b).

In this study, toluene—which accounted for 17.9% of the total BTEX composition among VOCs—was analyzed as a representative compound emitted from anthropogenic sources. Toluene readily reacts with photochemically generated hydroxyl (OH) radicals and degrades in the atmosphere. Its concentration increases in the presence of vehicle exhaust and is significantly elevated in areas where toluene is directly used, such as in industrial processes and solvent treatment [19]. In particular, high toluene concentrations were observed in zones heavily influenced by vehicle emissions. The average concentration measured in the Sasang Industrial Complex was 12.0 ± 35.8 µg/m^3^, with a maximum value of 380.26 µg/m^3^. As shown in Figure 4c,d, elevated concentrations were typically associated with areas of severe traffic congestion. In some locations where extremely high toluene levels were recorded, a vehicle maintenance shop was situated nearby. This suggests potential exposure from various oils and waste substances used in automotive servicing. In addition, two gas stations and one hydrogen charging station were located in close proximity to the high-concentration zones. These facilities are likely contributors to the observed toluene levels due to the release of volatile pollutants.

In the Sasang Industrial Complex, p-xylene accounted for 1.7% of the total BTEX composition among VOCs, indicating a relatively minor contribution. p-Xylene is commonly used as a raw material in the production of PET bottles and snack packaging. The average concentration of p-xylene measured in the study area was 1.2 ± 3.1 µg/m^3^, with a maximum observed value of 29.55 µg/m^3^. As illustrated in Figure 4e,f, although the section exhibited significant traffic congestion, no areas showed notably elevated p-xylene concentrations. Therefore, the correlation between traffic volume and p-xylene concentration appears to be weak.

In the Sasang Industrial Complex, m-xylene accounted for the highest proportion of BTEX compounds among the VOCs, comprising 45.9% of the total. According to the Ministry of Environment, xylene is widely used as a raw material in the production of basic chemicals and has a relatively high emission rate compared to other VOCs. Xylene is emitted not only from industrial facilities but also from construction sites using paints and adhesives and from vehicle exhaust, as it is a constituent of gasoline. The average m-xylene concentration measured in the study area was 30.7 ± 32.2 µg/m^3^, with a peak value of 230.93 µg/m^3^. As illustrated in Figure 4g,h, elevated concentrations were detected in areas with heavy traffic congestion and in zones where industrial facilities are concentrated. In particular, high m-xylene levels were observed near the Sasang Machinery Processing Complex, which houses small-scale operations involving hydraulic machinery, cylinder assembly, and metal polishing, suggesting these activities as likely emission sources. Additionally, nearby metal manufacturing and steel foundry plants located close to the roadside may contribute to emissions. A gas station also situated along the route likely adds to the overall m-xylene pollution burden in this area.

In the Sasang Industrial Complex, ethylbenzene accounted for 31.7% of the total BTEX concentration among VOCs. Ethylbenzene is commonly used as a solvent in the production of synthetic rubber, paints, adhesives, pesticides, and automobile fuels. At elevated concentrations, it can cause dizziness, and irritation in the eyes and the respiratory tract can occur. The average ethylbenzene concentration measured in the study area was 21.2 ± 18.3 µg/m^3^, with a maximum value of 122.04 µg/m^3^. As illustrated in Figure 4i,j, high concentrations were observed not only in zones with severe traffic congestion but also in areas where business establishments are densely clustered. Further analysis of the spatial distribution revealed that high ethylbenzene concentrations were often associated with metal manufacturing facilities—where metal cutting is actively performed—and with roadside steel foundries. In addition, several gas stations were located along the roadside, likely contributing to the elevated ethylbenzene levels.

### 3.4. Spatiotemporal Patterns and Pollution Hotspot Analysis of PM and VOC Concentrations in Saha Sinpyeong–Janglim Industrial Complex

In this study, the results of mobile measurements in the Saha Sinpyeong–Janglim Industrial Complex were visualized using a 3D GIS-based map. The GRIMM equipment was mounted on the SOF vehicle, with the sampling inlet positioned on the roof to ensure optimal air intake. Measurement data were synchronized with GPS coordinates and integrated into the GIS for spatial analysis.

**Figure 5:**
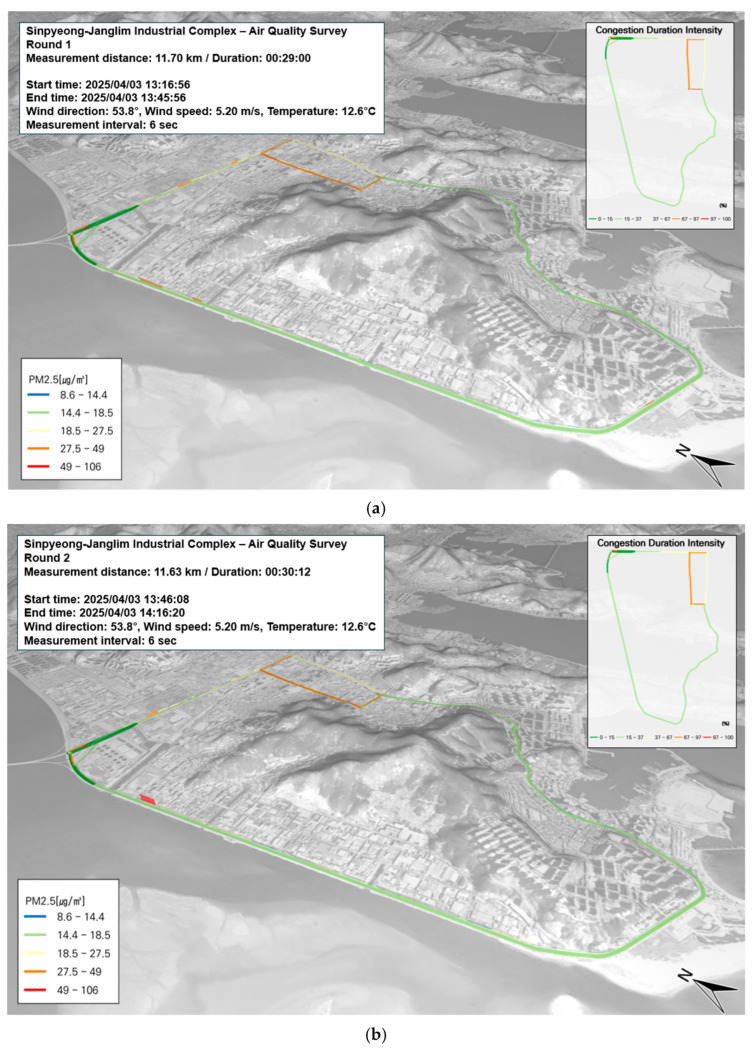

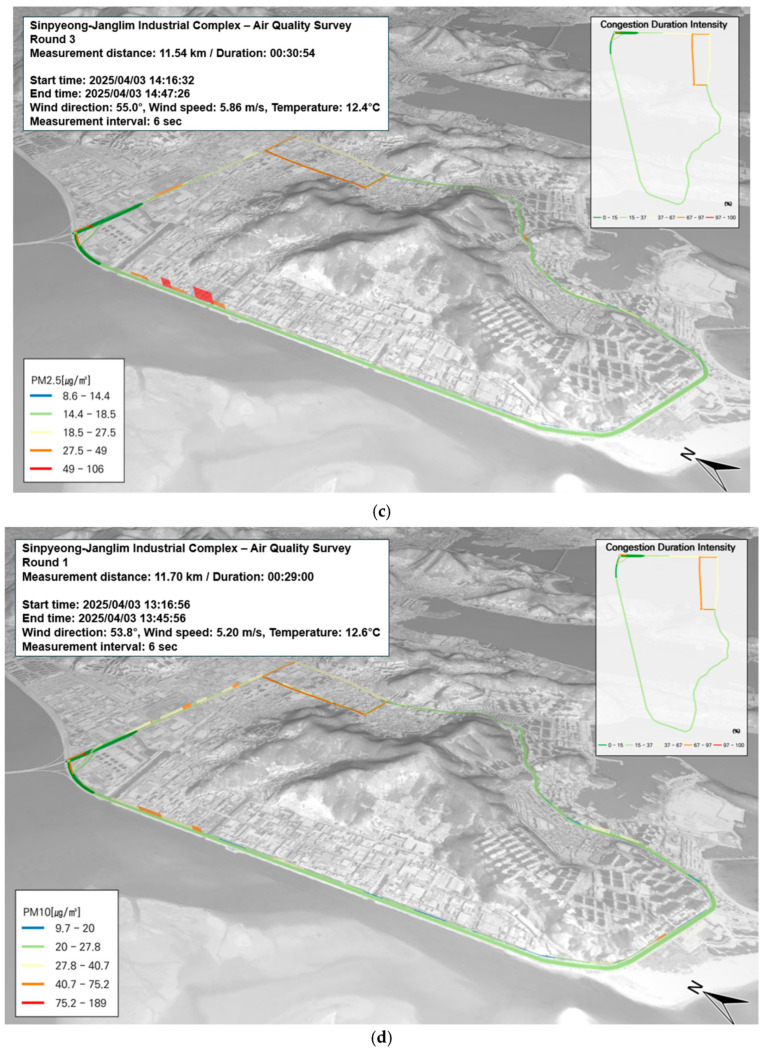

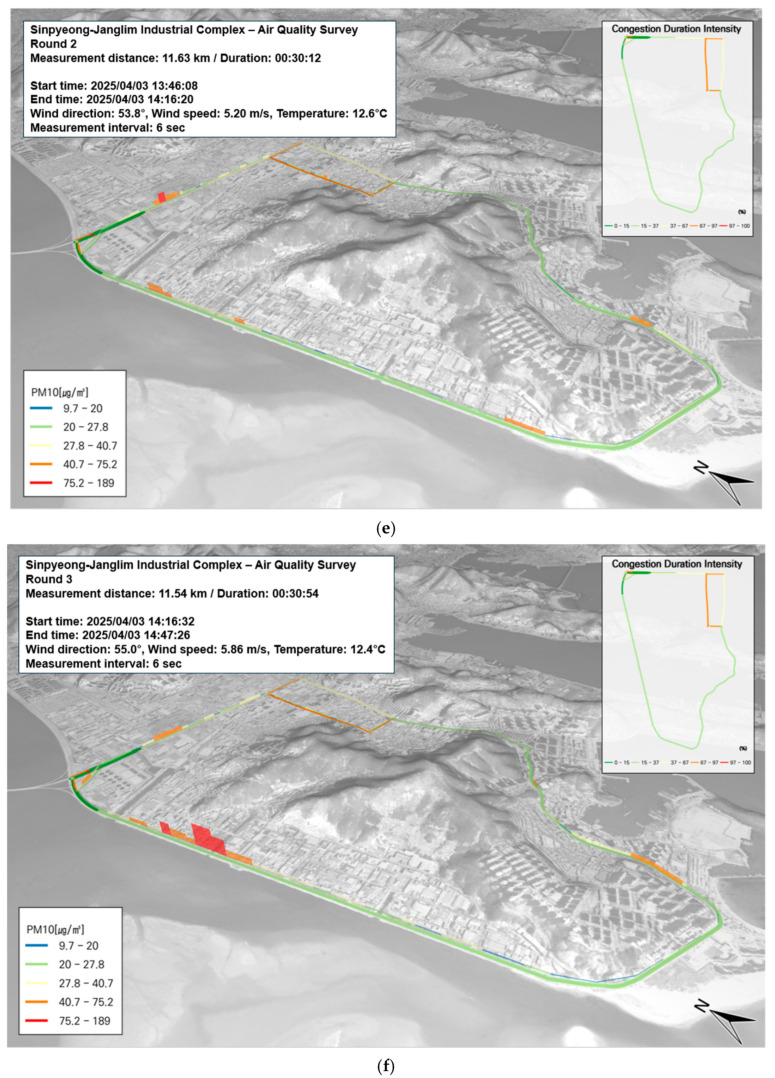
Spatial and temporal distribution of PM_2.5_ and PM_10_ concentrations on 3 April: (**a**–**c**) PM_2.5_, first to third runs; (**d**–**f**) PM_10_, first to third runs.

This study utilized GIS to derive results that reflect spatiotemporal characteristics according to time of day. In addition to the overall correlation analysis, we also examined the spatial and temporal patterns by mapping the data for each time period and location. PM_10_ and PM_2.5_ concentrations were recorded at 6 s intervals. Given the known correlation between fine particulate matter and road traffic, the level of congestion along the measurement path was analyzed to identify pollution hotspots. Although the overall traffic volume in the area was low, elevated PM concentrations were observed in certain segments, particularly those illustrated in Figure 5c,e,f. These areas were characterized by a high density of manufacturing facilities, including machinery and equipment plants, that were located immediately adjacent to the roadside. It is presumed that the elevated concentrations result from a combination of emissions from industrial activity and roadside vehicle operations, including gas station usage.

**Figure 6:**
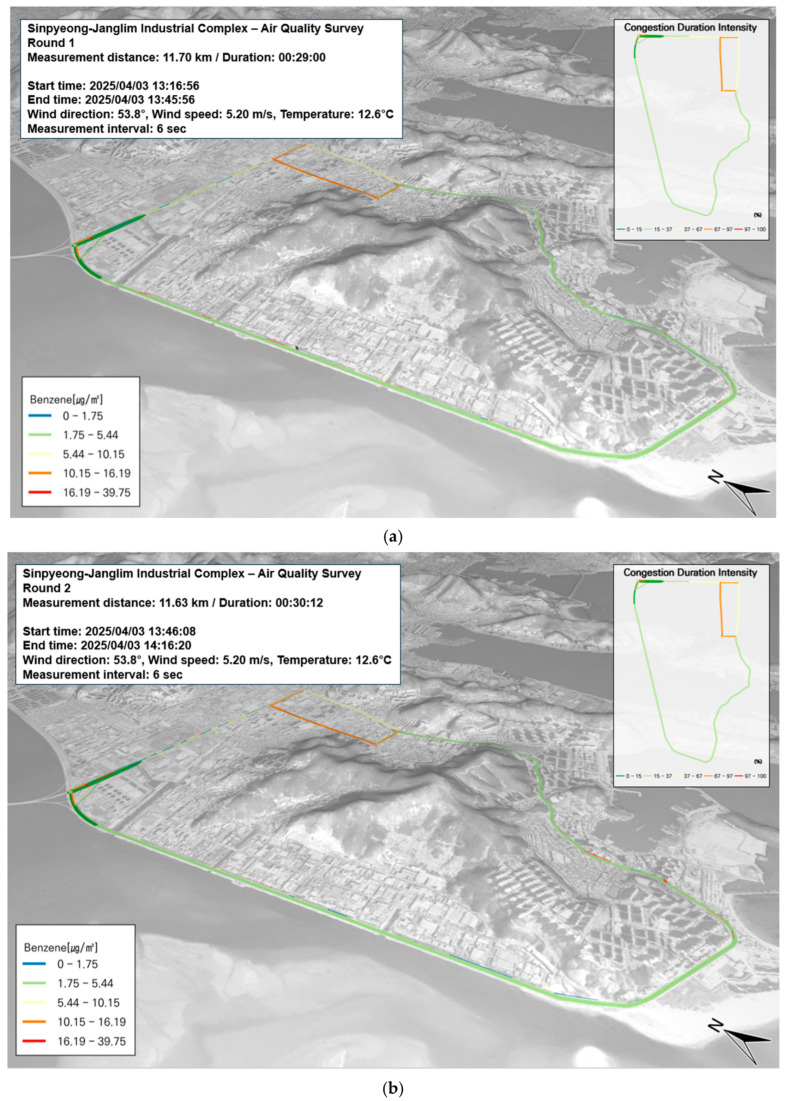

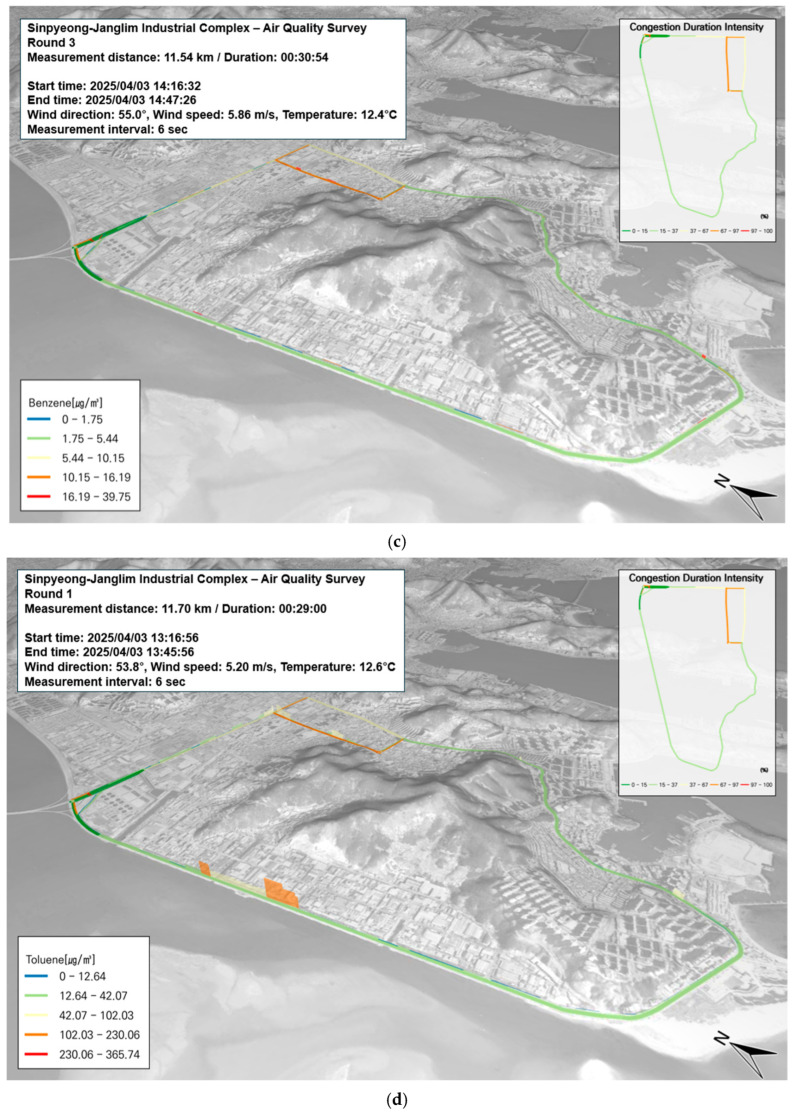

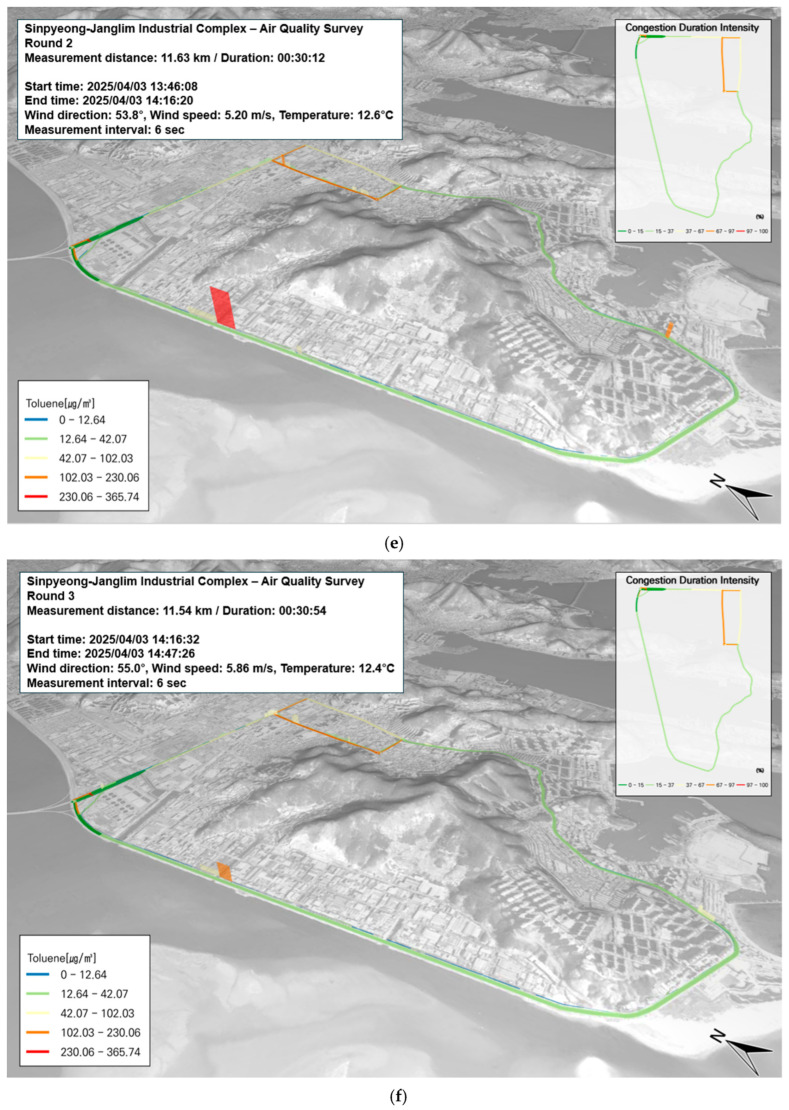

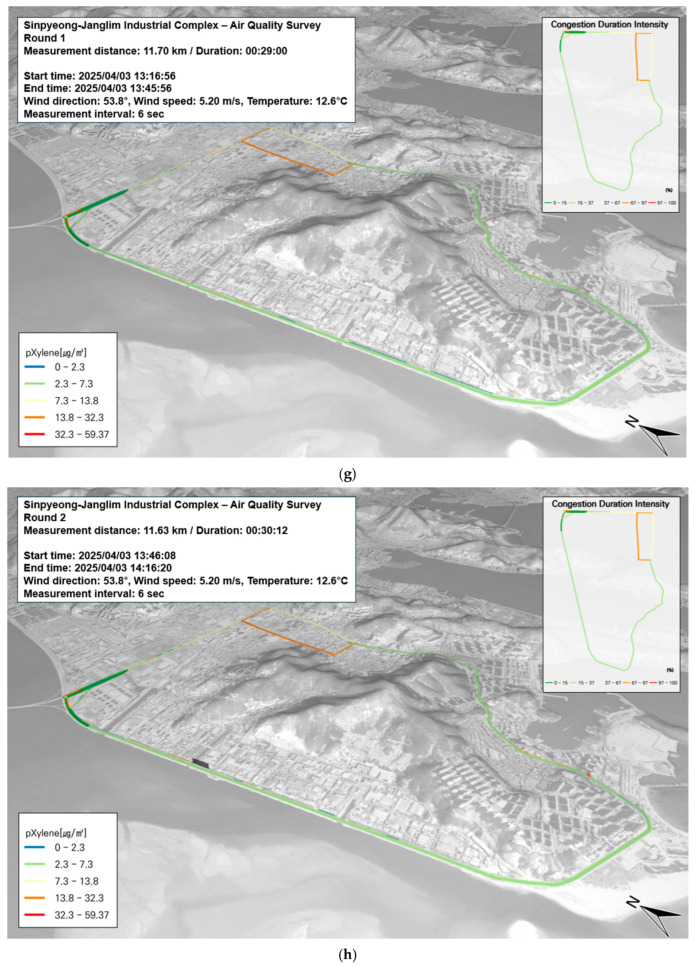

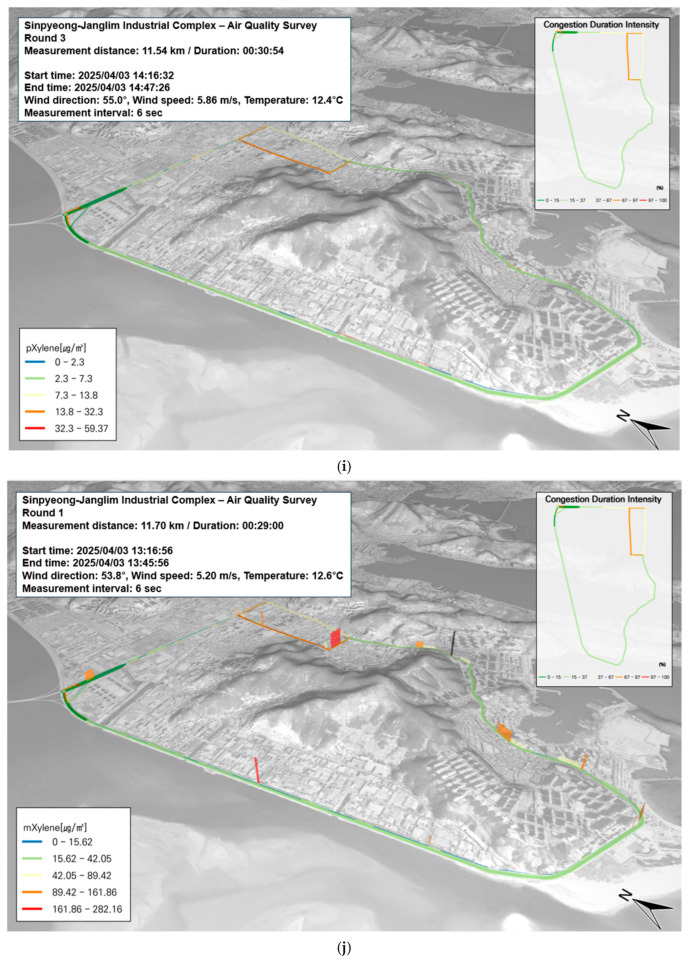

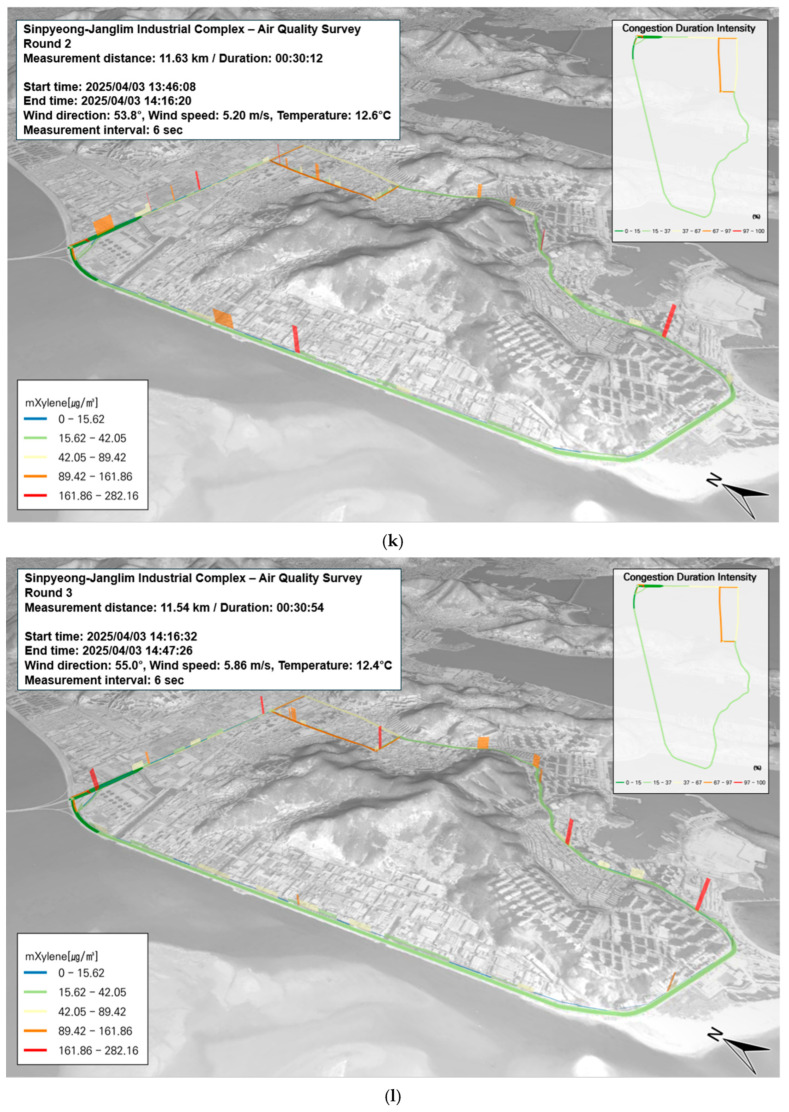

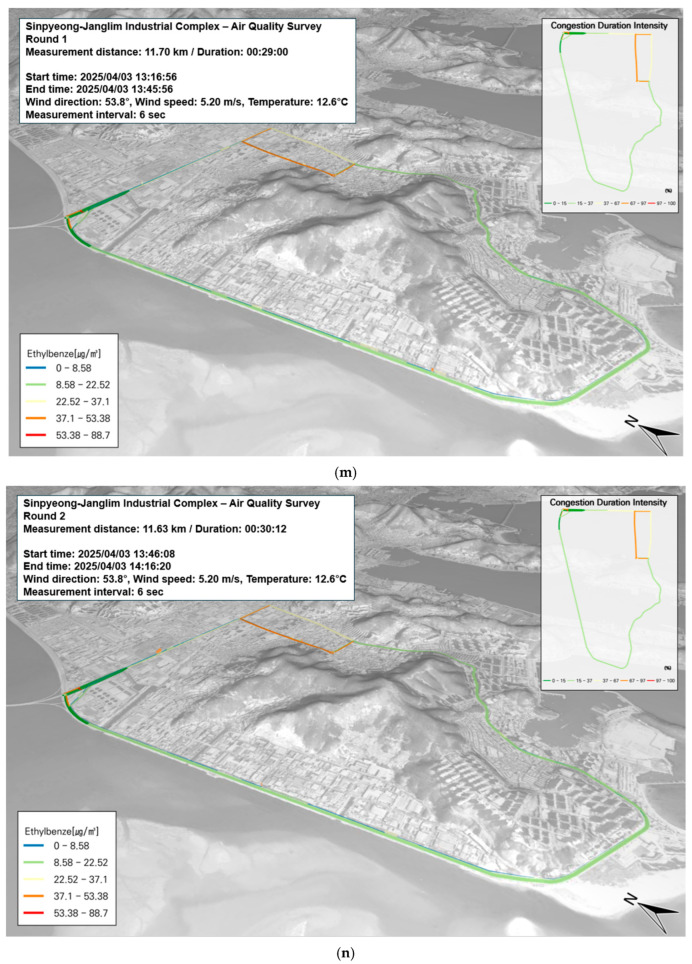

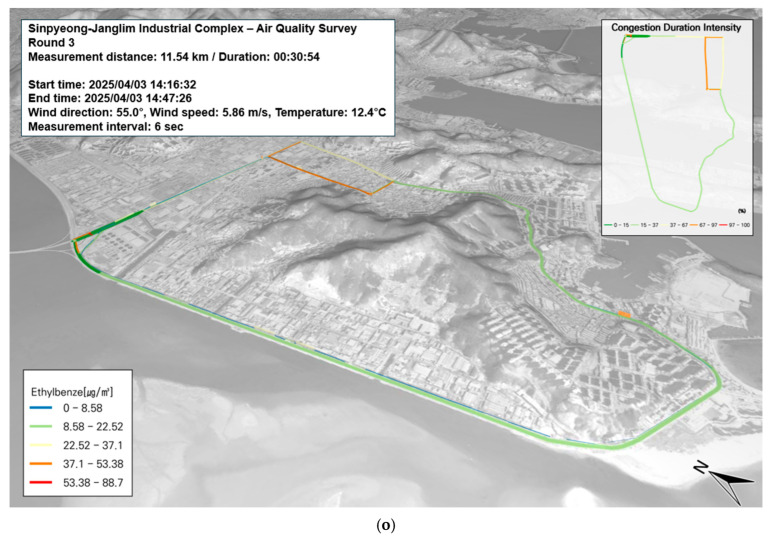
Temporal and spatial distribution of BTEX compounds (VOC) on 3 April: (**a**–**c**) benzene—first to third runs; (**d**–**f**) toluene—first to third runs; (**g**–**i**) p-Xylene—first to third runs; (**j**–**l**) m-Xylene—first to third runs; (**m**–**o**) ethylbenzene—first to third runs.

In the Saha Sinpyeong–Janglim Industrial Complex, benzene was selected as the representative BTEX compound for evaluation. The current air quality standard for benzene is set at an annual average of 1.5 ppb. Among all study areas, the highest benzene concentration was observed in the Sasang Industrial Complex during the measurement period, with a peak value of 39.75 µg/m^3^ and an average of 7.3 ± 5.9 µg/m^3^. In contrast, throughout the three measurement runs conducted in the Saha Sinpyeong–Janglim Complex, no locations exhibited relatively elevated concentrations. As shown in Figure 6a–c, benzene levels in this area appeared to be unrelated to road traffic congestion.

In the Saha Sinpyeong–Janglim Industrial Complex, toluene accounted for 25.5% of the total BTEX concentration among VOCs and was analyzed as a representative pollutant emitted from anthropogenic sources. The average toluene concentration measured in the study area was 15.6 ± 31.6 µg/m^3^, with a maximum value of 597.01 µg/m^3^. As shown in Figure 6d–f, the overall traffic congestion in the area was low. However, elevated toluene concentrations were observed in zones where machinery and equipment manufacturing facilities were densely clustered along the roadside. These concentrations are likely attributed to emissions from workplace processes, such as machinery fabrication and metal processing, rather than from vehicle traffic.

In the Saha Sinpyeong–Janglim Industrial Complex, p-xylene accounted for 9.4% of the total BTEX concentration among VOCs, indicating a relatively minor contribution. p-Xylene is commonly used as a raw material in the production of PET bottles and snack packaging. The average concentration of p-xylene measured in the study area was 5.7 ± 6.6 µg/m^3^, with a maximum observed value of 111.11 µg/m^3^. As shown in Figure 6g–i, the area exhibited low traffic congestion, and no segments showed relatively elevated p-xylene concentrations.

In the Saha Sinpyeong–Janglim Industrial Complex, m-xylene accounted for the highest proportion of BTEX among VOCs at 40.4%. According to the Ministry of Environment, xylene is widely used as a raw material for basic chemicals, and its emission levels tend to be higher than those of other VOCs. The average m-xylene concentration measured in the study area was 24.7 ± 42.1 µg/m^3^, with a maximum value of 295.97 µg/m^3^. As shown in Figure 6j–l, the area exhibited overall low traffic congestion. However, localized high concentrations were observed near areas with dense clusters of machinery manufacturing and processing facilities located adjacent to the roadside. These elevated levels are likely attributed to emissions from industrial activities rather than traffic. In particular, vehicle maintenance shops and textile manufacturing businesses located along the roadside appear to be significant contributors to m-xylene emissions in this area.

In the Saha Sinpyeong–Janglim Industrial Complex, ethylbenzene accounted for 12.7% of the total BTEX concentration among VOCs. Ethylbenzene is commonly used as a solvent in the production of synthetic rubber, paints, adhesives, pesticides, and automobile fuels. The average concentration measured in the study area was 7.8 ± 11.8 µg/m^3^, with a maximum value of 61.0 µg/m^3^. As shown in Figure 6m–o, the area exhibited generally low traffic congestion, and no specific locations exhibited relatively high ethylbenzene concentrations. These results suggest that the correlation between traffic volume and ethylbenzene levels in this area is low.

## 4. Discussion

There are approximately 1330 industrial complexes in Korea, many of which are located in close proximity to residential areas. As a result, environmental concerns such as odor dispersion, hazardous air pollutants (HAPs), and fine dust exposure frequently arise. Despite these risks, the availability of fixed air quality monitoring data within industrial zones remains limited, and public awareness of air toxics, such as volatile organic compounds (VOCs), is relatively low.

In this study, real-time mobile measurements were conducted in two representative industrial complexes in Busan—Sasang and Saha Sinpyeong–Janglim—to quantify BTEX compounds (benzene, toluene, ethylbenzene, and xylene) and particulate matter (PM_10_ and PM_2.5_) using Me-DOAS instruments mounted on a solar occultation flux (SOF) vehicle. Compared to traditional monitoring methods such as SIFT-MS and GC-MS/FID, which rely on fixed networks or offline sampling, Me-DOAS provides high-resolution, second-by-second VOC detection without sample pretreatment. This enables the spatial tracking of VOCs under varying environmental and traffic conditions.

Spatiotemporal distributions were analyzed by collecting mobile data along pre-defined routes traversing both residential and industrial areas. Data were synchronized with GPS and meteorological information, including wind direction and speed, and visualized using GIS-based 3D mapping. Although wind data were considered to interpret VOC patterns, this study did not include dispersion modeling or mixing height analysis. We acknowledge that these factors, along with temporal meteorological variability, may influence pollutant distributions and warrant consideration in future research. The survey was conducted during spring (April), when PM levels are typically elevated due to meteorological influences. This integrated approach allowed for the examination of relationships between VOC concentrations and variables such as traffic congestion, facility types, and spatial patterns. Although the mobile monitoring campaign was conducted over a limited period of two days in April—a season typically characterized by elevated PM concentrations due to meteorological factors—it may not fully represent long-term pollutant trends. However, unlike fixed monitoring stations, mobile measurements involve practical constraints such as vehicle and instrument rental, route scheduling, and real-time equipment operation. Despite these limitations, mobile monitoring offers valuable insights into spatial patterns and localized pollution hotspots, serving as a complementary tool to fixed monitoring networks. Future studies will aim to extend the monitoring duration across multiple seasons and integrate the results with conventional fixed-site monitoring data to enhance representativeness and temporal coverage.

In particular, with respect to the results of this study, the higher concentrations of xylene compared to benzene and toluene may be attributed to its widespread use in solvent-based industrial processes, such as painting, coating, and cleaning operations. Additionally, xylene’s lower volatility may lead to its accumulation under low-wind or stagnant conditions, resulting in elevated measured levels during mobile monitoring.

The correlation analysis showed a strong association between PM_10_ and PM_2.5_ at both sites (r = 0.848–0.894, *p* < 0.01), indicating the likelihood of shared emission sources. In the Sasang Industrial Complex, the correlation between toluene levels and traffic congestion was weak and statistically non-significant (r = 0.175, *p* > 0.05), suggesting a limited or incidental relationship. Elevated concentrations of toluene, m-xylene, and ethylbenzene were mainly detected near small-scale metal workshops, gas stations, and underground construction areas, pointing to localized emission sources. A significant correlation between benzene and m-xylene (r = 0.650, *p* < 0.01) suggests possible co-emission from common sources. In contrast, in the Saha Sinpyeong–Janglim area, p-xylene and toluene showed a moderate correlation (r = 0.442, *p* < 0.05). Overall, BTEX levels in this area appeared to be more strongly influenced by industrial land use and prevailing wind directions than by traffic activity. It is important to note, however, that confounding factors such as meteorological variability and temporal emission patterns may also have affected these relationships and should be considered in their interpretation.

While this study primarily examined the spatial patterns of VOC concentrations in relation to traffic and industrial activity, it is important to acknowledge the influence of meteorological and atmospheric chemical processes. Boundary layer dynamics, atmospheric stability, and air mass transport can significantly affect pollutant dispersion. Additionally, differences in VOC reactivity and atmospheric lifetime, particularly through reactions with hydroxyl radicals, play a critical role in determining their persistence and potential to form secondary pollutants. Future research should incorporate photochemical modeling and vertical mixing parameters to better elucidate the transformation and environmental behavior of VOCs in complex urban–industrial settings.

However, definitive source apportionment was beyond the scope of this study, as receptor modeling techniques, chemical tracers, or bivariate polar plots were not applied. Future research incorporating such methods would help to clarify the contributions of individual emission sources. GIS-based 3D visualization enabled the identification of VOC hotspots, improving interpretability over conventional 2D methods. By overlaying variables such as traffic data, facility type, pollutant concentrations, and meteorological conditions, this method supports more targeted air quality management strategies.

While Me-DOAS offers significant advantages in terms of real-time, high-resolution monitoring and ease of deployment, its limitations must also be acknowledged. Due to its optical path configuration, Me-DOAS can be sensitive to background signal noise, low SNRs (signal-to-noise ratios), and spectral overlaps—particularly for compounds such as toluene and xylene, which exhibit broad UV absorption features. To mitigate these issues, background correction was performed using low-concentration reference points, and outlier filtering procedures were applied. Benzene, with minimal spectral interference, proved to be most suitable for accurate detection. Despite these limitations, Me-DOAS remains a valuable tool for capturing dynamic VOC behavior in complex urban–industrial environments.

While the GIS-based 3D visualization effectively highlighted spatial emission patterns and localized VOC hotspots, we acknowledge that the choice of the interpolation method and the lack of semivariogram diagnostics may limit the ability to fully assess spatial uncertainty. Future work will incorporate formal interpolation algorithms such as Kriging or inverse distance weighting (IDW), along with semivariogram modeling, to improve the robustness of spatial analyses and uncertainty quantifications.

Additionally, we recognize that the use of 3D extrusions may visually exaggerate small concentration differences, potentially introducing perceptual bias. To address this, future visualizations will include normalization procedures or alternative cartographic techniques that more accurately reflect the underlying data distribution.

## 5. Conclusions

This study demonstrated the effectiveness of Me-DOAS-based mobile monitoring, integrated with GIS-based three-dimensional visualization, in characterizing the spatiotemporal distribution of VOCs in urban–industrial environments. By comparing two representative industrial complexes in Busan—Sasang, characterized by high traffic congestion, and Saha Sinpyeong–Janglim, with relatively lower traffic volume—we assessed the extent to which vehicular activity influences ambient VOC concentrations.

Correlation analysis indicated that the association between toluene levels and traffic congestion in Sasang was weak and statistically non-significant (r = 0.175, *p* > 0.05), suggesting that traffic alone is unlikely to be the predominant source. Instead, elevated concentrations of m-xylene and ethylbenzene were consistently observed near localized emission sources such as gas stations, underground construction sites, and small-scale metalworking facilities. In Saha, VOC levels were more closely linked to industrial zoning patterns and prevailing wind directions than to traffic activity. These findings imply that, although traffic may contribute to VOC variability, localized industrial operations and meteorological dispersion dynamics are more influential determinants of spatial concentration patterns.

Despite the temporal limitations of the monitoring campaign, the Me-DOAS system enabled high-resolution, real-time detection of BTEX compounds, with benzene yielding the most reliable results due to minimal spectral interference. The incorporation of GIS-based 3D visualization further enhanced spatial interpretability and enabled the identification of emission hotspots not captured by conventional fixed-site monitoring networks.

Overall, the study presents a scalable and data-driven framework for diagnosing local air pollution dynamics in complex industrial settings. Future research should prioritize multi-seasonal campaigns, integration of dispersion modeling and exposure assessment tools, and cross-validation with traditional monitoring methods to improve representativeness, reproducibility, and policy relevance.

## Figures and Tables

**Table 1:** Parameters measured and corresponding instruments used in this study. GPS: global positioning system; Me-DOAS: mobile extractive differential optical absorption spectroscopy; VOC: volatile organic compound.

Measurement	Instrument	Unit	Interval
Latitude and longitude	GPS	°	1 s
Elevation	GPS	m	-
Speed	GPS	km/h	-
VOCs (BTEX)	Me-DOAS	ppb	2 s
PM	Grimm 11-D	µg/m^3^	6 s
Wind speed	AWS	m/s	-

**Table 2:** VOC contaminants analyzed with Me-DOAS (mobile extractive differential optical absorption spectroscopy).

Compound	Formula	Molar Mass	Protonated Mass	Chemical Abstracts Service #
Benzene	C_6_H_6_	78.114	79.054	71-43-2
Toluene	C_7_H_8_	92.141	93.07	108-88-3
m-Xylene	C_8_H_10_	106.168	107.086	108-38-3
p-Xylene	C_8_H_10_	106.168	107.086	106-42-3
Ethylbenzene	C_8_H_10_	106.167	107.168	100-41-4

**Table 3:** Average concentrations of particulate matter (PM) and volatile organic compounds (VOCs) obtained from mobile monitoring.

Pollutant	Mean ± Standard Deviation (Unit: µg/m^3^, ppb)					
	2 April			3 April		
	Mean ± SD	Median	Percentage	Mean ± SD	Median	Percentage
PM_10_	20.7 ± 6.9	19.5	-	25.9 ± 12.5	23.4	-
PM_2.5_	15.2 ± 4.0	14.4	-	16.7 ± 6.4	15.4	-
Benzene	1.8 ± 2.7	0.4	2.7%	7.3 ± 5.9	6.8	12.0%
Toluene	12.0 ± 35.8	0.0	17.9%	15.6 ± 31.6	7.3	25.5%
p-Xylene	1.2 ± 3.1	0.0	1.7%	5.7 ± 6.6	4.5	9.4%
m-Xylene	30.7 ± 32.2	24.2	45.9%	24.7 ± 42.1	8.5	40.4%
Ethylbenzene	21.2 ± 18.3	19.9	31.7%	7.8 ± 11.8	0.0	12.7%

**Table 4:** Average concentrations of each air pollutant by measurement time interval.

Pollutant	Mean ± Standard Deviation (Unit: µg/m^3^, ppb)									
	2 April				3 April					
	1st		2nd		1st		2nd		3rd	
	Mean ± SD	Median	Mean ± SD	Median	Mean ± SD	Median	Mean ± SD	Median	Mean ± SD	Median
PM_10_	22.8 ± 7.9	21.4	19.0 ± 5.4	15.7	25.6 ± 7.7	23.9	24.6 ± 9.7	23.1	27.4 ± 17.5	23.2
PM_2.5_	16.5 ± 4.4	18.3	14.1 ± 3.1	13.7	17.2 ± 4.0	16.3	15.8 ± 5.0	14.7	17.3 ± 8.9	15.3
Benzene	2.4 ± 2.9	1.5	1.3 ± 2.4	0.0	7.1 ± 5.6	6.4	6.4 ± 5.8	5.5	8.4 ± 6.0	8.0
Toluene	13.6 ± 32.6	1.8	10.6 ± 38.2	0.0	21.6 ± 35.4	10.4	15.9 ± 38.5	7.7	9.6 ± 15.0	3.9
p-Xylene	1.7 ± 3.6	0.0	0.7 ± 2.4	0.0	5.4 ± 5.5	4.3	6.7 ± 8.3	5.4	5.1 ± 5.2	4.0
m-Xylene	29.4 ± 33.0	21.6	31.8 ± 31.6	26.0	19.0 ± 39.3	0.0	27.2 ± 45.1	11.8	27.4 ± 41.1	14.6
Ethylbenzene	19.9 ± 18.0	18.7	22.4 ± 18.5	21.2	7.3 ± 11.3	0.0	7.7 ± 11.6	0.0	8.2 ± 12.3	0.0

**Table 5:** Correlation between pollutants and traffic congestion in Sasang Industrial Complex.

	PM_10_	PM_2.5_	Benzene	Toluene	p-Xylene	m-Xylene	Ethylbenzene	Congestion Duration Intensity
PM_10_	1							
PM_2.5_	0.848	1						
Benzene	0.096	0.099	1					
Toluene	0.046	0.060	0.019	1				
p-Xylene	0.061	0.090	−0.121	0.109	1			
m-Xylene	0.029	0.015	0.650	−0.155	−0.236	1		
Ethylbenzene	0.198	0.234	0.109	0.118	−0.141	−0.016	1	
Congestion Duration Intensity	−0.014	−0.061	−0.026	0.175	−0.139	−0.094	−0.024	1

**Table 6:** Correlation between pollutants and traffic congestion in Saha Sinpyeong–Janglim Industrial Complex.

	PM_10_	PM_2.5_	Benzene	Toluene	p-Xylene	m-Xylene	Ethylbenzene	Congestion Duration Intensity
PM_10_	1							
PM_2.5_	0.894	1						
Benzene	−0.022	−0.009	1					
Toluene	0.116	0.100	0.192	1				
p-Xylene	0.096	0.103	−0.029	0.442	1			
m-Xylene	0.013	0.004	0.483	0.155	0.128	1		
Ethylbenzene	−0.116	−0.083	−0.083	−0.106	−0.252	−0.096	1	
Congestion Duration Intensity	−0.067	−0.047	−0.098	0.125	0.003	−0.010	0.028	1

## Data Availability

The original contributions presented in this study are included in the article. Further inquiries can be directed to the corresponding authors.

## References

[1] (2024). Korea Industrial Complex Corporation (KICOX). https://www.kicox.or.kr/index.do.

[2] World Health Organization (WHO) (2000). Air Quality Guidelines for Europe.

[3] Che J.S., Che J.M., Jeon J.M., Kang B.W., Kim J.H., Moon K.J., Park K.T., Kang D.I. (2024). The study on emission characteristics of gas-phase hazardous air pollutants generated at the large-scale industrial complexes. J. Korean Soc. Atmos. Environ..

[4] Lee T.J., Lee S.M., Chae J.S., Jeon J.M., Kim D.S., Jo Y.M. (2021). Inventory of ozone precursor VOCs from organic solvents used in residential workplaces and assessment of ozone formation contribution. J. Korean Soc. Atmos. Environ..

[5] Seo Y.K., Chung S.H., Baek S.O. (2011). Current status and prospective of hazardous VOC in ambient air. J. Korean Soc. Atmos. Environ..

[6] Ministry of Environment (2023). Clean Air Conservation ACT. https://elaw.klri.re.kr/eng_service/lawView.do?lang=ENG&hseq=41386.

[7] Shin S.H., Park J.S., Kim J.B., Kim P.H., Kim C.H., Hwang K.C., Park S.M., Lee J.Y., Park J.M., Kim J.H. (2024). Characteristic of volatile organic compounds distribution in downtown Ansan near industrial complex. J. Environ. Anal. Health Toxicol..

[8] Ministry of Environment (2024). National Air Pollutants Emission 2012.

[9] Che J.S., Lee S.M., Jeon J.M., Hong J.H., Lee C.H., Ham S.W., Park J.H., Seo K.A., Kang D.I., Jang K.W. (2024). Emission characteristics of hazardous air pollutants in industries subject to domestic fugitive emissions reduction. J. Korean Soc. Atmos. Environ..

[10] Yoom M.R., Jo H.J., Kim G.B., Chang J.Y., Lee C.W., Lee B.E. (2021). Exposure to PAHs and VOCs in residents near the sinpyeong Jangrim industrial complex. J. Environ. Health Sci..

[11] Hwang K.C., An J.G., Lee S.H., Choi W.S., Yim U.H. (2020). A Study on the Ozone Formation Potential of Volatile Organic Compounds in Busan using SIFT-MS. J. Korean Soc. Atmos. Environ..

[12] Choe H.S., Jeon W.B., Kim D.J., Yang C.Y., Mun J.H., Park J.H. (2023). Analyzing the changes in O3 concentration due to reduction in emissions in a metropolitan area: A case study of Busan during the summer of 2019. J. Environ. Sci. Int..

[13] Im Y.S., Yoo S.J. (2012). Study on the Emission Characteristics of Odor Compounds from Industrial Complex in the Sasang District of Busan.

[14] Cheong J.P., You S.J. (2011). Characteristics and identification of ambient VOCs Sources in Busan industrial area. Korean Soc. Environ. Eng..

[15] Kim A.Y., Lee H.M., Lee J.Y., Kim Y.P. (2024). Policy directions for the volatile organic compounds in Seoul and Busan, Korea. Part. Aerosol Res..

[16] Min J.H., Kim B.G., Ju H.J., Kim N.Y., Hwang Y.S., Lee S.H., Hong Y.S. (2024). Analysis of the association between air pollutant distribution and mobile sources in Busan using spatial analysis. J. Environ. Health Sci..

[17] Ju H.J., Lee S.H., Min J.H., Hwang Y.S., Hong Y.S. (2023). Exploration of an area with high concentrations of particulate matter and biomonitoring survey of volatile organic compounds among the residents. J. Environ. Health Sci..

[18] Kim Y.J., Shin H.J., Lee H.Y., Lee J.H., Choi J.H., Jeong Y.J. (2024). Health risk assessment of residents’ exposure to air pollutants around the Sinpyeong-Jangrim Insudtrial complex in Busan. Toxics.

[19] Maantay J.A., Tu J.C., Maroko A.R. (2009). Loose-coupling an air dispersion model and a geographic information system (GIS) for studying air pollution and asthma in the Bronx, new York city. Int. J. Environ. Res. Public Health.

[20] Hasan M.M., Mahamud M., Dewan A., Ahmed F., Rahman M. (2022). Spatial and temporal analysis of impacts of hurricane florence on air quality using real-time mobile monitoring. Int. J. Environ. Res. Public Health.

[21] Shuaibu N.S., Qin C., Chu F., Ismail B.B., Ibrahim A.M., Indabawa M.G., Abdalmohammed S.A.A., Zhao G., Wang X. (2024). Traceability tagging of volatile organic compound sources and their contributions to ozone formation in Suzhou using vehicle-based portable single-photon ionization mass spectrometry. Environ. Sci. Eur..

[22] Rutherford M., Koss A., de Gouw J. (2024). Mobile VOC measurements in Commerce City, CO reveal the emissions from different sources. J. Air Waste Manag. Assoc..

[23] Dang A.J., Kreisberg N.M., Cargill T.L., Chen J.-H., Hornitschek S., Hutheesing R., Turner J.R., Williams B.J. (2024). Development of a Multichannel Organics In situ enviRonmental Analyzer (MOIRA) for mobile measurements of volatile organic compounds. Atmos. Meas. Tech..

[24] Huang Y., Che X., Jin D., Xiu G., Duan L., Wu Y., Gao S., Duan Y., Fu Q. (2022). Mobile monitoring of VOCs and source identification using two direct-inlet MSs in a large fine and petroleum chemical industrial park. Sci. Total Environ..

[25] Healy R.M., Sofowote U.M., Wang J.M., Chen Q., Todd A. (2022). Spatially Resolved Source Apportionment of Industrial VOCs Using a Mobile Monitoring Platform. Atmosphere.

[26] Liang Q., Bao X., Sun Q., Zhang Q., Zou X., Huang C., Shen C., Chu Y. (2020). Imaging VOC distribution in cities and tracing VOC emission sources with a novel mobile proton transfer reaction mass spectrometer. Environ. Pollut..

[27] Kim M.K., Kim D.H., Seo J.Y., Park D.S. (2024). GIS-based analysis of volatile organic compounds in bucheon, korea, using mobile laboratory and proton-transfer-reaction time-of-flight mass spectrometry methods. Toxics.

[28] Kim M.K., Kim D.H., Jang Y.L., Lee J.Y., Ko S.W., Kim K.H., Park C.S., Park D.S. (2023). Determination of the spatial distribution of air pollutants in Bucheon, Republic of Korea, in winter using a GIS-based mobile laboratory. Toxics.

[29] Youn S.J., Jo K.H., Kim H.S., Song G.B., Lee S.B., Jeong J.Y. (2020). Measurement of Hazardous Air Pollutants in Industrial Complex Using Mobile Measurement System with SIFT-MS. J. Korean Soc. Atmos. Environ..

[30] FluxSense AB (2017). Using Solar Occultation Flux and Other Optical Remote Sensing Methods to Measure VOC Emissions from a Variety of Stationary Sources in the South Coast Air Basin.

[31] Johansson J., Mellqvist J. Quantification of Industrial Emission of VOCs NO2 and SO2 by SOF and Mobile DOAS. FINAL REPORT AQRP Project 10-006. https://research.chalmers.se/publication/540618/file/540618_Fulltext.pdf.

